# Assessing suffering of patients on cancer treatment and of those no longer treated using ESAS–Total Care (TC)

**DOI:** 10.1007/s00520-023-08035-4

**Published:** 2023-09-16

**Authors:** Guido Miccinesi, Carla Ripamonti, Silvia Leoni, Monica Gandelli, Patricia Di Pede, Vania Visani, Paolo Ambrosini, Giulia De Feo, Laura Bellandi, Luisa Toffolatti, Cosimo Chelazzi, Consuelo Trinci, Francesca Chiesi

**Affiliations:** 1 Clinical Epidemiology Unit, Oncological Network Prevention and Research Institute (ISPRO), Florence, Italy; 2 Palliative Medicine, Department of Medical and Surgical Specialties, Radiological Sciences and Public Health, Universita’ degli Studi di Brescia, Brescia, Italy; 3 Fondazione ANT Italia ONLUS, Bologna, Italy; 4 Internistic and Geriatric Supportive Care Unit, Fondazione IRCCS, Istituto Nazionale dei Tumori di Milano, Milan, Italy; 5 Department of Medical Oncology, IRCCS Fondazione Istituto Nazionale dei Tumori, Milan, Italy; 6 Department of Neuroscience, Psychology, Drug, and Child’s Health (NEUROFARBA), Section of Psychology, University of Florence, Florence, Italy

**Keywords:** Total care, Oncologic treatments, Home palliative care, Terminal cancer, Psychometric validation, Financial, Isolation, Spirituality

## Abstract

**Aim:**

The aim of the study was to assess the suffering of patients on oncologic treatment and of those no longer on treatment. Preliminarily, we aimed to confirm the psychometric properties of Edmonton Symptom Assessment System–Total Care (ESAS-TC) in different stages of the disease. The ESAS-TC screens physical and psychological symptoms, but also spiritual pain, discomfort deriving from financial problems associated with illness, and suffering related to social isolation.

**Methods:**

A sample of consecutive advanced cancer patients on oncologic therapies treated at the Internistic and Geriatric Supportive Care Unit (IGSCU) of Istituto Nazionale dei Tumori, Milano, and of terminal patients no longer on treatment and cared for by the Fondazione ANT palliative home care team were asked to fill the ESAS-TC. In order to strengthen the previous validation study of the ESAS-TC, 3-ULS (to assess social isolation), JSWBS (to assess spiritual well-being), COST-IT (to assess financial distress), and KPS (to assess functional status) were administered too.

**Results:**

The questionnaires were self-reported by 108 patients on treatment (52% >60 years old, female 53%, and 61% with KPS 90–100) and by 94 home care patients (71% >60 years old, female 51%, and 68% with KPS 10–50). The sound psychometric characteristics of ESAS-TC were confirmed. Patients on treatment showed lower total ESAS-TC score (19.3 vs 52.7, *p*<.001) after controlling for age and functional status, and lower financial distress (*p*.<001). Financial distress, spiritual suffering, and social isolation, after controlling for age, showed a significantly higher score in home care patients.

**Conclusions:**

Only through an adequate routine assessment with validated tools is it possible to detect total suffering, the “Total pain” of patients, and treat it through a multidisciplinary approach. The study confirms the reliability and validity of the Italian version of ESAS-TC and the importance of supportive and early palliative care fully integrated with oncological treatment.

## Introduction

The assessment of pain, physical and psychological symptoms, and toxicities caused by oncological therapies that generate a large part of the patient’s suffering is or should be routine in clinical practice. The continuous and timely use of patient-reported outcomes (PROs) has been shown to significantly improve the quality of life and reduce emergency room visits and hospitalization of cancer patients [1–10].

The Edmonton Symptom Assessment System (ESAS) [11] is a Patient Reported Outcome Measure (PROM) already widely used as screening and longitudinal monitoring of the most frequent symptoms in many care settings including palliative care, supportive care, oncology, nephrology, and other disciplines in both inpatient and out-patient settings [11–14].

The Edmonton Symptom Assessment System–Total Care (ESAS-TC) is a PROM recently modified and validated in Italian language [15] with the aim of expanding the information obtained through the original ESAS [11] to social, financial, and spiritual distress [16–30]. The previous validation study [15] was conducted mainly in patients undergoing cancer treatments, or on follow-up. Thus, some items/symptoms were less endorsed (i.e., low frequencies were observed in physical symptoms such as nausea, dyspnea, loss of appetite, and for financial toxicity) and this aspect might limit the generalizability of the results on the psychometric properties of the ESAS-TC and the use of the scale in different stages of disease and/or in different settings of care.

Considering this, the aim of the current study was to compare patients still on oncologic treatment, with those no more oncologic treatment, to highlight differences in spiritual suffering, distress due to financial problems, social isolation, and ESAS-TC total score, in order to acquire knowledge about the multifaceted patient’s suffering which will allow to further advance towards a tailored and total care, adapted to the stage of the disease. We included patients undergoing cancer treatment in relapse and/or with metastases (i.e., advanced cancer), and home palliative care patients who have stopped the cancer therapies for the terminal stage of their disease.

To conduct the study, we aimed to preliminary confirm the psychometric and clinical value of the ESAS-TC in patients at different stages of disease.

## Patients and methods

From July 2022 until November 2022, all the consecutive out-patients undergoing cancer treatment that referred to the dedicated Internistic and Geriatric Supportive Care Unit (IGSCU) of Fondazione IRCCS, Istituto Nazionale dei Tumori (INT) of Milano [31] and the consecutive patients cared for by Florence home palliative care team of Fondazione ANT Italia, Onlus, who were not treated with oncologic therapies due to the terminal stage of the disease, were recruited for the study, once they gave informed consent to the participation and to the use of personal data.

Fondazione ANT is a non-profit provider of palliative care which follows patients who can no longer be treated with oncological therapies and for whom the oncologists make a specific request for home care because of the terminal stage of the disease. Moreover Fondazione ANT provides supportive care to patients undergoing oncological treatments or during the follow-up so as to allow the patients to continue their oncological therapies or to deal with other therapies in good clinical and psychosocial conditions. Among the support, there is the possibility to evaluate and treat not only the physical symptoms but also to speak with the pastoral counselor, with the psychologist, and social worker and to have more time for ongoing care before accessing home care for patients considered terminally ill. For this study, patients who passed directly from oncological care to home care without an intermediate period of supportive care were selected.

Fondazione ANT has been engaged since 1985 in free home care for cancer sufferers, in palliative care training for medical and nursing staff, and in research and prevention against cancer. The purpose of the dedicated Internistic and Geriatric Supportive Care Unit (IGSCU) is to treat patients with symptoms related to cancer therapies, who require hydrations, transfusions, and all the medical therapies needed during cancer treatments and to provide psychosocial and spiritual support according to the needs of each individual patient [31]. Approval was obtained from the local Ethics Committee of the INT (Prot. 279/21) and for ANT Foundation from the Comitato Etico Regionale per la Sperimentazione Clinica della Regione Toscana Sezione: AREA VASTA CENTRO (21/649-0ss).

### Main inclusion criteria

All the patients were included in the study if they met the following inclusion criteria:

- Certified histological diagnosis of cancer
- Age >18 years
- Being treated with anti-cancer therapies and undergoing supportive care at IGSCU of INT

OR

- Being cared for by home palliative care team, in the terminal phase of the disease, not on oncological treatment, and who did not receive any previous supportive care
- Health conditions that did not affect the subject’s ability to complete questionnaires independently, and to release personal information through interviewing by staff
- Absence of cognitive impairments
- Signed informed consent

Patients who did not meet the above inclusion criteria were excluded.

The investigating physicians of the IGSCU and those of the Fondazione ANT explained to the patients the aims and the methods of the study, collecting any questions and doubts. After having signed the informed consent, clinical data were recorded for all study participants. For terminally ill patients, the self-reported questionnaires were administered in the first week of home care.

At IGSCU of INT, advanced cancer patients were consecutively enrolled at the first referral or during the forthcoming supportive medical treatment.

Specifically, information about age, gender, education, marital status, profession, and religious believe/practice was collected. Clinical data evaluated by the researcher included the performance status, the primary tumor, the stage of the disease, the type of oncological treatment in progress, and the presence of comorbidities. Then, the investigating physician provided the patient the paper-based questionnaires that were self-filled by patients. The medical staff was available for patients in case further doubts raised during the compilation.

The questionnaires were the following.

**Fig. 1:**
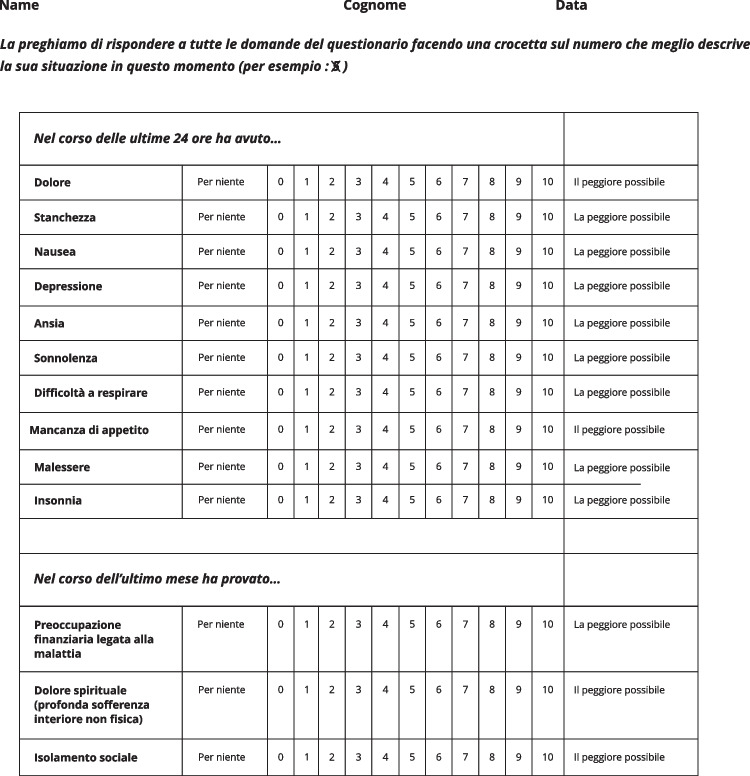
Edmonton Symptom Assessment System–Total Care (ESAS-TC). English translation of the three additional items: Preoccupazione finanziaria legata alla malattia :  Worries  due to financial problems associated with  illness; Dolore spirituale (profonda sofferenza interiore non fisica):  Spiritual pain; Isolamento sociale: Social isolation

-The *Edmonton Symptom Assessment System–Total Care* (*ESAS-TC*) Italian version [15]—reported in Fig. 1—consists of 13 items describing symptoms that the patient rates in intensity on a 0 to 10 numerical scale, with 0 representing “no symptom” and 10 the “worst possible symptom”; patients were asked to refer to the previous 24 h when answering to the first 10 items, while they had to refer to the last month for the last 3 items. The ESAS-TC includes the original ESAS scale [11] that allows the assessment of 10 different symptoms (pain, fatigue, nausea, depression, anxiety, drowsiness, distress, lack of appetite, difficulties in breathing, insomnia) and three additional items related to financial distress, spiritual suffering, and social isolation. The original 10 items questionnaire was validated psychometrically in Italian among patients undergoing cancer treatment or follow-up [32].

In the legend of Fig. 1, we added the English translation of the three additional items of the ESAS-TC questionnaire.

-*Comprehensive Score for financial Toxicity* (*COST*) [25, 26]. As part of the functional assessment of chronic disease therapy (FACIT) measurement system, the scale measures disease-related financial toxicity [25, 26] and the Italian version [27] consisting of 11 items on a 5-point Likert scale from 0 to 4 (not at all - a little - quite - a lot). The total score obtained by adding the scores of the individual items varies from 0 to 44 (the last item is not included in the calculation) and higher scores correspond to lower toxicity.

-*Three-Item UCLA Loneliness Scale* (*3-ULS*) [33]. This is a very short scale used to rate feelings of loneliness or social isolation derived from the Revised UCLA Loneliness Scale [34]. Each question is rated on a 3-point scale: 1 = Hardly ever; 2 = Sometimes; 3 = Often. All items are added together to give a total score. Higher scores indicate greater levels of loneliness.

-*Jarel Spiritual Well-Being Scale* (*JSWBS*) [35]. In its original version, it is composed of 21 items, on a 5-point Likert Scale from 1= strongly disagree to 5 = strongly agree, divided into three factors (Faith and belief, Life and responsibility, Satisfaction and fulfillment in life) [35]. For the Italian version [36], the data analysis shows that the scale has good psychometric properties, but the confirmatory factor analysis highlights some differences in the structure compared to the original version, probably due to cultural differences between Americans and Italians, which required a new factorial solution and the exclusion of some items. Then, a scale composed of 16 items divided into three factors was proposed: Faith and belief (e.g., “Prayer is an important part of my life”), Meaning of Life (e.g., “I find meaning and meaning in my life”), and Quality of relationships (e.g., “I am able to receive and give love to others”).

-The *Karnofsky Performance Status* (KPS) was described for the first time in 1949 by Karnofsky and Burhcenal [37]. It evaluates a patient’s functional status as a comprehensive 11-point scale correlating to percentage values ranging from 100 (no evidence of disease, no symptoms) to 0 (death). This scale was able to predict prognosis and it is useful for defining the purpose of therapies and determining the care planning.

## Statistical analysis

### Sample size

The collected sample size was necessary in order to conduct factor analyses based on the ratio of the number of cases (*N*) and the number of variables/item (*p*) which must be as follows: *N*/*p* ≥ 10. In this case, 130 patients were sufficient. However, given the 11-point rating of the items, a sample size of 200 cases was considered to ensure adequate statistical power for data analysis [38].

For the correlational analyses, the required sample size was determined on the expected effect size [39]. Convergent validity is adequate if we observe correlations with a large effect size (i.e., ≥ .55) [40]. Therefore, approximately 70 cases with complete data were needed (*p*<.001, *β*=.95).

For group comparisons, a large effect size was expected (Cohen’s *f* ≥ .40). Thus, the total sample size should consist of 180 cases (*p*<.001, *β*=.95) [41].

### Analysis strategy

We decided to compare patient suffering as measured by ESAS-TC between two consecutive sample of patients in different stages of the disease and therefore treated in different settings: at the IGSCU (out-patients still in treatment) and at home (patients no longer in cancer treatment).

Preliminarily, we confirmed the psychometric characteristics of the recently validated Italian questionnaire ESAS-TC. Item descriptives were computed to verify departures from normal distributions (values outside the range of −1 and 1 were considered indicators of non-normal distributions) [42]. To determine the underlying structure of the scales, an exploratory factor analysis was conducted on FACTOR [43]. This analysis implements a re-sampling (bootstrap) procedure that allows robust estimations for any estimate of interest (e.g., factor loadings, fit indices). Number of factors was determined using parallel analysis based on minimum rank factor analysis. To evaluate the model residuals, we used the weighted root mean square residual (WRMR), with the relative 95% confidence intervals. Expected values of WRMR for an acceptable model is < 1.0. McDonald’s Omega coefficient was used for internal consistency. McDonald’s values *ω* ≥ .70 are considered satisfactory. Construct validity was tested using Spearman’s correlations among the ESAS-TC spiritual suffering, financial distress, and social isolation items with the COST, JSWBS, and ULS-3 scores. Strong correlations were expected (0.55 or higher).

To compare advanced (on cancer treatment) and terminal (no cancer treatment) patients, several analyses were carried out. Analyses of covariance (ANCOVA) were performed to assess the effect of the stage of the disease on the ESAS-TC total score, spiritual suffering, financial distress, social isolation, controlling for KPS scores, and age. The partial eta squared (*ηp*^2^) was used for the effect size (values lower than 0.06 suggest a small effect, values from 0.06 to 0.14 a medium effect, values from 0.14 a large effect). Additionally, to further control a possible confounding effect of age on social isolation, *t*-tests were used to compare treatment and no treatment patients in three different age groups (≤60 years, 61–70 years, and 71–85 years, respectively). Cohen’s *d* values from 0.2 to 0.5 are indicators of a small effect, values from 0.5 to 0.8 represent a medium effect, and values from 0.8 a large effect. Finally, ANCOVA was used to assess the effect of the stage of the disease and religious believe/practice on the spiritual suffering, controlling for the KPS score and age.

## Results

### Sample description

The ESAS-TC and the other scales were administered to 202 patients (51.5% women) aging from 29 to 92 years (mean = 67.70, SD =12.31, median = 68). Socio-demographic and clinical data are reported in Table 1.

**Table 1:** Sample socio-demographic and clinical characteristics by treatment

	*ON ONCOLOGIC* *TREATMENT (INT)* *N=108*		*NO ONCOLOGIC* *TREATMENT (ANT)* *N=94*		*TOTAL SAMPLE* *N=202*	
	*N*	*%*	*N*	*%*	*N*	*%*
Gender						
Female	57	52.8	47	50.0	104	51.5
Male	51	47.2	47	50.0	98	48.5
Age						
≤40 years	6	5.6	1	1.1	7	3.5
41–60 years	45	41.7	6	6.4	51	25.2
61–70 years	34	31.5	24	25.5	58	28.7
71–85 years	23	21.3	63	67.0	86	42.6
Educational level						
Primary school	7	6.5	23	24.5	30	14.9
Secondary school	20	18.5	27	28.7	47	23.3
High school	53	49.1	26	27.7	79	39.1
University	28	25.9	18	19.1	46	22.8
Marital status						
Single	22	20.6	6	6.5	28	14.0
Married	72	67.3	60	64.5	132	66.0
Divorced	4	3.7	6	6.5	10	5.0
Widowed	9	8.4	21	22.6	30	15.0
Occupation						
Self employed	18	16.7	10	10.6	28	13.9
Salaried worker	37	34.3	24	25.5	61	30.2
Unemployed	1	0.9	3	3.2	4	2.0
Retired	43	39.8	49	52.1	92	45.5
Housewife	4	3.7	6	6.4	10	5.0
On furlough	2	1.9	2	2.1	4	2.0
Other	3	2.8	0	0.0	3	1.0
Religious believe/practice						
Practicing believer	36	33.3	34	36.2	70	34.8
No practicing believer	55	50.9	38	40.4	93	46.3
No believer	16	14.9	22	23.4	36	18.9
Phase of the disease						
Advanced						
Relapse +	97	89.8	0	0.0	97	48.0
Metastatic	11	10.2	33	35.1	44	21.8
Terminal	0	0.0	61	64.9	61	30.2
Type of tumor						
Solid	78	72.2	86	94.5	164	82.4
Hematologic	30	27.8	5	5.5	35	17.6
Diagnosis						
Breast	27	25.0	5	5.7	32	16.4
Lung	8	7.4	29	33.3	37	19.0
Colon	5	4.6	9	10.3	14	7.2
Prostate	4	3.7	6	6.9	10	5.1
Sarcoma	5	4.6	1	1.1	6	3.1
Pancreas	1	0.9	5	5.7	6	3.1
Head and neck	13	12.0	4	4.6	17	8.7
Hodgkin lymphoma	3	2.8	1	1.1	4	2.1
Non-Hodgkin lymphoma	14	13.0	1	1.1	15	7.7
Leukemia	5	4.6	3	3.4	8	4.1
Myeloma	10	9.3	0	0.0	10	5.1
Gastric	5	4.6	6	6.9	11	5.6
Hepatocellular	1	0.9	3	3.4	4	2.1
Endometrial	1	0.9	3	3.4	4	2.1
Kidney	3	2.8	1	1.1	4	2.1
Other	1	0.9	2	2.3	3	1.5
Therapy*						
Chemotherapy	54	50.0	0	0.0	54	27.0
Radiation therapy	14	13.0	0	0.0	14	6.9
Hormonal	28	25.9	0	0.0	28	13.9
Targeted therapy	8	7.4	0	0.0	8	4.0
Immunotherapy	10	9.3	0	0.0	10	5.0
Experimental	8	7.4	0	0.0	8	4.0
Surgical	5	4.6	0	0.0	5	2.5
Other	2	1.9	0	0.0	2	1.0
Comorbidity*						
Hypertension	29	26.9	22	23.9	51	25.4
Heart disease	10	9.3	22	23.9	32	16.0
Osteoarticular	4	3.7	3	3.2	7	3.5
Diabetes	7	6.5	10	10.8	17	8.5
Neurological	1	0.9	5	5.6	6	3.0
Other	49	45.4	37	39.4	86	42.6
Karnofsky performance status						
10–50	1	0.9	64	68.1	65	32.2
60–80	41	38.0	29	30.9	70	34.7
90–100	66	61.1	1	1.1	67	33.2

*Some patients received more than one therapy and have more than one comorbidity

Thus, reported frequencies are number of affirmative answers and the relative percentage on the total sample

### Preliminary psychometric analyses

Descriptive analysis of each ESAS item showed that answers were spread along the 11 response options, but high frequencies were observed for the 0 answer. Thus, some values of Skewness and Kurtosis were higher than 1, indicating positive asymmetric and/or leptokurtic distributions. Means ranged from 1.32 to 4.53 and standard deviations from 2.32 to 3.17. All indices are reported in Table 2.

**Table 2:** Descriptives, factor loadings, and reliability indices for the 13 items of the Edmonton Symptom Assessment System–Total Care (ESAS-TC)

	*Mean*	*Standard deviation*	*Skewness*	*Kurtosis*	*Factor loading (95%CI)*	*Item test correlation*	*McDonald’s ω if item dropped*
Item							
1	2.71	2.81	0.72	−0.62	0.68 (0.57; 0.75)	.60	.90
2	4.53	2.93	−0.01	−1.05	0.82 (0.76; 0.87)	.75	.90
3	1.41	2.32	1.58	1.31	0.67 (0.58; 0.75)	.52	.90
4	2.73	3.04	0.64	−0.99	0.87 (0.83; 0.91)	.78	.89
5	2.61	2.80	0.75	−0.63	0.77 (0.70; 0.82)	.68	.90
6	3.35	3.10	0.48	−1.02	0.68 (0.59; 0.74)	.58	.90
7	1.76	2.62	1.28	0.29	0.60 (0.50; 0.69)	.48	.91
8	2.87	3.17	0.66	−0.90	0.77 (0.71; 0.83)	.68	.90
9	3.43	3.10	0.34	−1.17	0.91 (0.87; 0.93)	.84	.89
10	2.02	2.62	1.05	−0.18	0.53 (0.41; 0.62)	.46	.91
11	2.26	2.84	0.91	−0.53	0.55 (0.45; 0.65)	.46	.91
12	2.50	3.05	0.96	−.040	0.77 (0.68; 0.82)	.68	.90
13	1.32	2.96	0.94	−0.43	0.69 (0.61; 0.77)	.58	.90

Given the ordinal and non-normal distributions of the data, we used the polychoric correlations and the Robust Unweighted Least Squares (RULS) estimation method for factor analysis. The unidimensional structure of the ESAS-TC was confirmed by parallel analysis, and it explained the 56% of the variance. The WRMR was 0.070 (.95%CI: .064–.075) representing good fit of the one-factor model. Factor loading ranged from .53 to .91 (all values and the relative 95% confidence interval are reported in Table 2).

For internal consistency, McDonald’s Omega was excellent (.91; 95%CI: .89–.93). No increases in alpha values were observed if any of the individual items were removed from the scale and item–total correlation values ranged from .46 to .84.

For construct validity, we observed very large correlations between the COST total score and the financial distress item (*r*(*N*=189) = .65 (95%CI: .55;.73), *p*<.001), and the ULS-3 total scores and the social isolation item (*r*(*N*=202) = .64 (95%CI: .54;.73), *p*<.001). Conversely, a moderate correlation was found between the ESAS-TC spiritual suffering item and the JSWS Meaning of Life scale score (*r*(*N*=198) = −.36 (95%CI: −.49;−.23), *p*<.001), along with no significant correlations with the JSWS Faith and belief scale score (*r*(*N*=190) = .12 (95%CI: −.03;.25), *p*= .10) and the JSWS Quality of relationships scale score (*r*(*N*=197) = −.12 (95%CI: −.27;.02), *p*=.11). Not surprisingly, the latter scale correlated negatively and moderately with the ESAS-TC social isolation item (*r*(*N*=197) = −.44 (95%CI: −.54; −.33), *p*<.001).

### Comparing treatment (*N*=108) vs no treatment (*N*=94) groups

ESAS-TC score differences were observed (*F*(1, 198)= 13.95, *p* < .001, *ηp*^2^= .07) after controlling the effect of age (*F*(1, 198)= 4.55, *p* < .05, *ηp*^2^= .02) and KPS (*F*(1, 198)= 20.52, *p* < .001, *ηp*^2^= .10). Similarly, a significant difference was found in financial distress (*F*(1, 201)= 12.36, *p* < .001, *ηp*^2^= .06) after controlling only the effect of age (*F*(1, 201)= 14.02, *p* < .001, *ηp*^2^= .07), while the KPS effect was not significant (*F*(1, 201)= 2.03, *p* = .15, *ηp*^2^= .01). Since this result could be related to the patient’s employment, we checked if the two samples differed in occupation, but no differences were observed (*χ*2[6, *N*=202] = 8.92, *p*=.18, see Table 1 for descriptives).

**Fig. 2:**
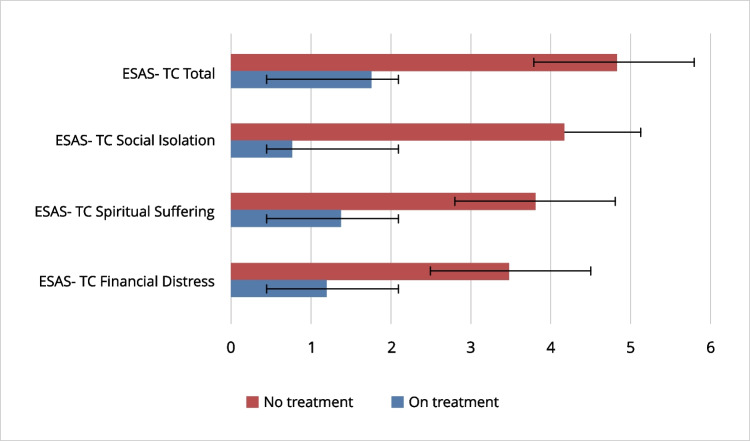
Differences in financial distress, spiritual suffering, social isolation items, and total score (divided by the scale number of items) of the Edmonton Symptom Assessment System–Total Care (ESAS-TC) by treatment

Treatment and no treatment groups reported different levels of spiritual suffering (*F*(1, 201)= 12.87, *p* < .001, *ηp*^2^= .06) after controlling the effect of age (*F*(1, 201)= 5.05, *p* < .05, *ηp*^2^= .03) given the non-significant effect of the KPS score (*F*(1, 201)= 0.41, *p* = .52, *ηp*^2^= .002). Finally, results showed a significant difference in social isolation (*F*(1, 201)= 18.58, *p* < .001, *ηp*^2^= .09) and the effects of age and KPS were not significant (*F*(1, 201)= 1.06, *p* = .31, *ηp*^2^= .01, *F*(1, 201)= 2.84, *p* = .09, *ηp*^2^= .01, respectively). For each variable, the no treatment group scored significantly higher than on treatment patients (Fig. 2).

To better investigate the absence of a relationship between age and social isolation, we conducted *t*-tests between treatment and no treatment patients of each age group (see Table 1). Results showed a significant and very large difference in social isolation scores in each age group (*t*(56)= −3.11, *p* < .01, *d*= −1.25, *t*(56)= −8.41, *p* < .001, *d*= −2.24, and *t*(84)= −4.61, *p* < .001, *d*= −1.12, respectively, from the younger to the older group). The no treatment group scored significantly higher than treatment patients across ages (Group ≤60: *M*_*Treatment*_= 1.06, *SD*= 2.17, *M*_*NoTreatment*_= 4.00, *SD*= 3.51; Group 61–70: *M*_*Treatment*_= 0.29, *SD*= 0.84, *M*_*NoTreatment*_= 4.63, *SD*= 2.84; Group 71–85: *M*_*Treatment*_= 0.83, *SD*= 2.96, *M*_*NoTreatment*_= 3.95, *SD*= 2.96).

**Fig. 3:**
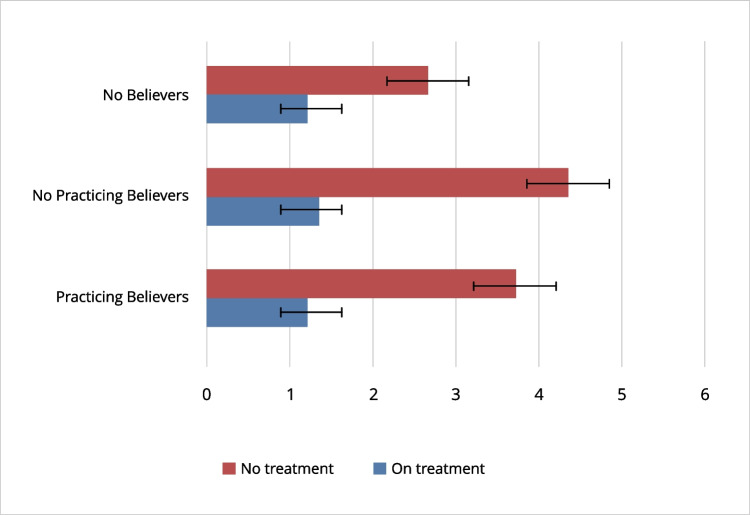
Differences in spiritual suffering of the Edmonton Symptom Assessment System–Total Care (ESAS-TC) by treatment and religious believe/practice

Finally, after controlling the effect of age (*F*(1,200)= 6.17, *p* < .001, *ηp*^2^= .02), the ANCOVA revealed a significant differences in spiritual suffering (*F*(1, 200)= 8.91, *p* < .01, *ηp*^2^= .04) between patient groups, while no differences related to religious believe/practice were observed (*F*(1, 200)= 1.46, *p* =.23, *ηp*^2^= .01). The interaction effect was not found (*F*(1,200)= 1.68, *p* = .19, *ηp*^2^= .02). Specifically, the no treatment group scored significantly higher than treatment patients across practicing believer, no practicing believer, no believer groups (Fig. 3).

## Discussion

The current study aimed at acquiring knowledge about the multifaceted patient’s suffering to further advance towards a tailored and total care of cancer patients.

Why is it important to use ESAS-TC and validate it in other languages? While ESAS [11] is a PROM suitable for the assessment of physical and emotional symptoms, it is known that it is necessary to evaluate the patient’s global suffering such as loneliness (social isolation) [44], worries, and distress due to financial problems related to the disease [27].

Furthermore, many data in the literature indicate the patient’s need for spirituality and how much this can influence the patient’s quality of life [18, 19].

ESAS is a simple tool to which the assessments of other problems/concerns/sufferings can be added to understand other factors that cause suffering to the patient and contribute to the so-called TOTAL PAIN [45] highlighted by Cicely Saunders [46] more than 50 years ago.

In the present study, we included patients undergoing cancer treatment in advanced stage of disease and patients in the first week of home care due to terminal stage of disease. To accomplish this goal, preliminarily, the study provided further evidence of the psychometric strength of the ESAS-TC to support its use with these cancer patients for a total assessment of physical and emotional symptoms.

Specifically, compared to the previous ESAS-TC validation study [15], we obtained higher endorsement percentages for physical symptoms (e.g., nausea, loss of appetite), financial toxicity, and for some psychological symptoms (e.g., depression). Given these different response rates, we confirmed the one-dimension structure and the good reliability of the ESAS-TC [15], as well as the soundness of three items added to the ESAS to develop the ESAS-TC: the spiritual suffering (i.e., a deep soul pain that can be an important component of cancer patient distress), the financial distress (i.e., patients’ feelings about their financial condition, including perceived economic well-being and income adequacy after the diagnosis and treatments), and the social isolation (i.e., the loneliness experienced by the patient that misses the social support to face illness). All together, these findings provide evidence for the generalizability of the results on the psychometric properties of the ESAS-TC, and they allow the use of the scale in different stages of disease and/or in different settings of care.

Nonetheless, it is still difficult for the health care professionals as well as for the informal caregivers to understand the depth and the extension of this total or comprehensive suffering intimately experienced by each patient, because it is difficult to be expressed through words. The correct use of PROMs like ESAS-TC comes to help and sustain in this difficult task both to diagnose, to monitor, and to detect the efficacy of the interventions. Indeed, it is crucial that the tool is simple and sound, and this is the reason why preliminarily we confirmed in different settings and in different stages of the disease the psychometrically soundness of the ESAS-TC, making it effectively recommendable for all cancer patients.

An important result is the high score of suffering due to physical and emotional symptoms as well as in new “total” dimension of social isolation, financial distress, and spiritual pain among home palliative care patients. Those patients never underwent an early palliative and/or supportive care during anti-cancer treatment prior entering the home care program. Their suffering, evaluated during the first week, was high and significantly higher than that reported by patients referred to IGSCU of INT. A possible explanation is that the lack of continuity of care through supportive/palliative care and the transition from cancer therapy (thought of by the patient as a phase of curable cancer) to home care (terminal disease) with the detachment from the doctors and nurses who have always followed them during hospitalization or in ambulatory setting may be the reason of the spiritual distress, social isolation, and the intensity of symptoms in the patients’ first week of home care.

Fortunately, patients referred to IGSCU of INT, even if undergoing active oncological therapies, which may by itself lead to suffering, were well supported by a dedicated internist/geriatric supportive care unit that provided both medical and multi-dimensional interventions to reduce the suffering.

This confirms the importance of supportive care and early palliative care fully integrated with oncologic treatment [47, 48]. However, also this approach may be not enough if the evaluation is limited to physical symptoms. The deep suffering of the patient as well as the spiritual and financial distress that does not fall within the interest rates of health professionals risks not being considered within the “Total Pain” and therefore not being listened to and treated during all stages of illness [45, 46].

The multi-dimensional suffering of patients, including spiritual, social, and financial issues, needs to be actively assessed by the health professionals, to provide a comprehensive treatment. Indeed, in the context of a life-limiting, complex disease like cancer, the shift from active hospital treatment to home palliative care can be associated with growing social isolation possibly due to the setting and the presence of symptoms that may limit social interactions (such as fatigue), certainly with spiritual issues that may be related to the loss of hope or meanings, denial, difficult coping, and fear of death.

Although the treatments are free, home patients express higher financial distress than those on hospital treatment. As a tentative explanation, we can presume that these patients understand that they can no longer be a financial support for their loved ones, and this may explain their concern related to the economic domain.

Moreover, the result that spiritual suffering does depend on clinical condition but does not depend on religious status (believer practicing, believer not practicing, not believer) is not a minor one. It confirms the consensus that has been affirming in Medicine during the last 10 years about the necessity of a clear distinction between religiosity and spirituality and the prevalence of the last term, which refers to an universal human dimension, and which is commonly meant to indicate the personal and universal search for meaning and connectedness rather than any specific belief, faith, or religious ritual [18, 19]. Moreover, ESAS-TC can help identifying which component of this “Total Pain” is more relevant for each single patient and tailor or monitor interventions in a more personalized and consistent way, adding quality to the care of patients and potentially contributing to lower their suffering and to increase their quality of life. Additionally, one big strength of the ESAS-TC is its simplicity which helped patients to self-report on those cumbersome issues without adding the stress of expressing themselves in complicated ways.

A limit of the study is that patients were only oncological, with a clear cut-off between active treatment and palliative care, while in many other clinical scenarios this distinction is not well defined since patients can stay on disease-specific therapy until the end (e.g., patients with heart failure). To overcome this limit, furthermore, multicentric studies considering broader clinical scenarios and samples of multicultural patients are advised to strengthen the results of this study.

## Conclusion

Our results show the need and opportunity for a direct and standardized assessment of patients by validated tools to diagnose and monitor the patient’s multi-dimensional suffering with the aim to detect the efficacy of multi-modal interventions.

The ESAS-TC confirms to be a useful tool to capture the suffering of cancer patients that goes beyond physical and emotional also in advanced and terminal stages and in different settings of care and will allow us all to further advance towards an integral care capacity (Total Care).

Bringing further empirical evidence in favor of the validity of the ESAS-TC, we intend to strengthen the solidity of the tool and the possibility of using it in contexts other than the one in which it was initially validated.

## Data Availability

The datasets used and/or analyzed during the current study are available from the corresponding author on reasonable request
